# The roles of endolithic fungi in bioerosion and disease in marine ecosystems. I. General concepts

**DOI:** 10.1080/21501203.2017.1352049

**Published:** 2017-07-27

**Authors:** Frank H. Gleason, Geoffrey M Gadd, John I Pitt, Anthony W. D Larkum

**Affiliations:** a School of Life and Environmental Sciences, University of Sydney, Sydney, NSW, Australia; b Geomicrobiology Group, School of Life Sciences, University of Dundee, Dundee, Scotland; c Food, Safety and Quality, CSIRO, Ryde, NSW, Australia

**Keywords:** Calcareous substrates, calcium carbonate, coral skeletons, diseases of corals, zooxanthellae, global climate change

## Abstract

Endolithic true fungi and fungus-like microorganisms penetrate calcareous substrates formed by living organisms, cause significant bioerosion and are involved in diseases of many host animals in marine ecosystems. A theoretical interactive model for the ecology of reef-building corals is proposed in this review. This model includes five principle partners that exist in a dynamic equilibrium: polyps of a colonial coelenterate, endosymbiotic zooxanthellae, endolithic algae (that penetrate coral skeletons), endolithic fungi (that attack the endolithic algae, the zooxanthellae and the polyps) and prokaryotic and eukaryotic microorganisms (which live in the coral mucus). Endolithic fungi and fungus-like boring microorganisms are important components of the marine calcium carbonate cycle because they actively contribute to the biodegradation of shells of animals composed of calcium carbonate and calcareous geological substrates.

## Introduction

### Importance of this research topic

During the past three decades, the prevalence and the rate of transmission of emerging infectious diseases, and the frequency of epizootics increased significantly in both terrestrial and aquatic ecosystems, primarily due to social, demographic and environmental transformations (Wilcox and Gubler 2005; Fisher et al. 2012; Burge et al. 2013). It is extremely important to thoroughly understand host–parasite interactions in these times of environmental and climate change so that better management practices for preserving both wild and cultivated species and species diversity can be designed and implemented. Species of endolithic fungi are known to cause significant bioerosion and diseases of many host animals in marine ecosystems. Unfortunately, our knowledge of the ecological functions of these microorganisms is only superficial.

This and the subsequent reviews focus on current knowledge of true fungal, algal, stramenopilian (eukaryotic) and cyanobacterial (prokaryotic) endolithic parasites in marine environments and their ecological functions. The basic concepts of ecology related to rock penetrating microorganisms including lichens and mycorrhizal fungi must be discussed first. Rock penetrating or rock boring microorganisms can be divided into two groups: those which penetrate calcareous substrates formed by living organisms and those which penetrate substrates formed by geological processes. Endoliths offer excellent model systems for the study of the interaction between physical and biological factors in microbial ecology, geobiology and astrobiology.

### A theoretical interactive model for the ecology reef-building corals

According to Bentis et al. (2000), reef-building corals appear to exist in dynamic equilibrium with four principal partners: (1) interconnected polyps of a colonial coelenterate, (2) endosymbiotic dinoflagellate zooxanthellae residing in the host’s endoderm, (3) endolithic algae that penetrate coral skeletons and (4) endolithic fungi that attack one or more of the endolithic algae, the zooxanthellae and the polyps. In our opinion, the collection of prokaryotic and eukaryotic microorganisms in the coral mucus needs to be included as a partner as well (number 5) (Table 1). These can be either beneficial or harmful (Harel et al. 2008). In this revised model, the five principal partners are actually populations including many different genotypes. The composition and interactions of these five partners are controlled by environmental factors.

**Table 1.** Interactions between components of the bottom part of a generalised coral reef food web.

Partners	Site	Trophic type	Trophic level	Direction of energy flow
1) Interconnected polyps	Inside Skeleton	Heterotrophic	Primary consumer	From external food, Zooxanthellae and Endolithic Algae to coral tissues
2) Zooxanthellae	Endoderm Polyp Tissue	Autotrophic	Producer	Provides carbon nutrients for polyp tissues
3) Endolithic algae	Within skeleton	Autotrophic	Producer	Provides carbon nutrients for polyp tissues and Endolithic fungi
4) Endolithic fungi	Within skeleton	Heterotrophic	Primary consumer	From endolithic algae and proteins inside the skeleton
5) Fungi and bacteria in coral mucus	Outside skeleton	Heterotrophic	Primary consumers	From secreted nutrients or from coral tissue when parasitic
6) Zooxanthellae	Free-living in water	Autotrophic	Producers	Provides nutrients for parasites and predators when released
7) Parasitic dinoflagellates	Free-living in water	Heterotrophic	Primary consumers	From living and atrophied zooxanthellae when released from coral tissues
8) Zooplankton protists and small animal predators	Free-living in water	Heterotrophic	Primary or secondary consumers	From all dinoflagellates, other phytoplankton and fungi
9) Endolithic algae In environmentoutside corals	Calcareous sediments /substrates	Autotrophic	Producers	Provides nutrients for Parasites and Predators
10) Endolithic fungiin coral reefenvironment	Calcareous sediments/ substrates	Heterotrophic	Primary consumer	From endolithic algae and proteins inside calcareous structures

Finally, the dynamics of populations of heterotrophic dinoflagellate parasites of the zooxanthellae, other protists and small animals which are parasites, predators or grazers on any of the other partners, as well as their predators (numbers 6, 7 and 8), and endolithic fungi and algae in the environment outside corals (numbers 9 and 10) needs to be considered as parts of this model. This extends the model to include the entire coral reef food web (Table 1).

Kendrick et al. (1982) isolated into pure culture a number of bioeroding fungi from the interior of the aragonite skeleton of living corals in the Caribbean and South Pacific, most of which were dikaryomycotan anamorphs. These fungi are thought to be a major cause of bioerosion in coral reef ecosystems. Although fungi undoubtedly play many important ecological roles in coral ecosystems, they have been largely ignored in the past (Bentis et al. 2000). Our current and very limited knowledge of the roles of fungi in corals and coral reef ecosystems has been reviewed in detail by Raghukumar and Ravindran (2012). Corals, lichens and mycorrhizae are all symbiotic relationships involving fungi.

### Primary objectives of this review

Many species of true fungi, fungus-like microorganisms and algae are known to bore into solid rock, sand grains and shells. In this review, we discuss what is known about the different types of rock penetrating endoliths with emphasis on marine species of true fungi which bore into corals and briefly describe their morphology, life history, mechanisms of infection, general roles in ecology, host substrate interactions, participation in the marine calcium carbonate cycle and the possible effects of global climate change on growth.

## Characteristics of rock transforming fungi

Fungi are significant agents of geochemical change in the environment and capable of numerous transformations of metals and minerals (Gadd 2007, 2010). They can therefore contribute to the structural and chemical alteration of rocks, and mineral-based substrates including those produced biogenically. The study of the roles of fungi in geologically relevant processes, such as metal and mineral transformations, can be termed geomycology, an important part of the more general area of geomicrobiology (Gadd 2007, 2010). In the terrestrial environment, such processes are important in rock bioweathering, contributing to the formation and development of mineral soil, and global biogeochemical cycles for component elements, including their availability to living organisms (Sterflinger 2000; Burford et al. 2003; Gadd 2007). In aerobic terrestrial environments, free-living and symbiotic fungi are of great importance, especially when considering rock surfaces, soil and the plant root–soil interface. While fungi are also ubiquitous in freshwater and marine ecosystems, as important decomposers and pathogens, their geomicrobial significance is rather unappreciated in such locations in comparison with prokaryotes (Gadd 2008). However, it is now known that they have a significant presence in locations not usually regarded as prime fungal habitats, e.g. acid mine drainage, deep aquatic sediments, hydrothermal vents and the igneous oceanic crust (Reitner et al. 2006; Gorbushina 2007; Vázquez-Campos et al. 2014; Iversson et al. 2016). In such locations, fungi may exist in symbiosis with chemolithotrophic prokaryotes (Ivarsson et al. 2016). The ecological success and geoactive properties of fungi are underpinned by their growth habit and metabolism, and their ability to form symbiotic relationships with other organisms, such as lichens and mycorrhizas. Lichens are a fungal growth form, consisting of a symbiotic partnership between a fungus and a photosynthetic organism, either a eukaryotic alga (in the green algal family Trebouxiaceae) or a cyanobacterium and sometimes both (Purvis and Pawlik-Skowronska 2008). It is now known that they can also contain a yeast as another fungal partner (Spribille et al. 2016). Lichens are pioneer colonisers of rocks, and initiators of bioweathering biofilms that are involved in the early stages of mineral soil formation. They are ubiquitous in the terrestrial environment and can be extremely tolerant of extreme environmental conditions. Symbiotic root-associated mycorrhizal fungi are associated with approximately 80% of plant species and are responsible for major mineral transformations and redistributions of inorganic nutrients, such as essential metals and phosphate, as well as carbon flow through the ecosystem. The activities of mycorrhizal fungi can lead to changes in the physicochemical characteristics of the root environment and enhanced weathering of soil minerals, resulting in metal and phosphate release (Gadd 2007). Ectomycorrhizal mycelia may respond to the presence of different soil silicate and phosphate minerals (apatite, quartz and potassium feldspar) by regulating their growth and activity, for example, colonisation, carbon allocation and substrate acidification (Rosling et al. 2004a, 2004b). As well as the many kinds of free-living fungi found on rock substrates, many of which may be of soil origin, a particular group of fungi inhabiting rock substrates are the microcolonial fungi (MCF). These do not exhibit the filamentous hyphal mode of growth but produce unicellular yeast-like and microcolonial growth, occurring as small black melanised colonies resulting in dark brown to black discolouration on colonised surfaces (Marvasi et al. 2012). This growth habit confers a high degree of resistance to environmental stress (Gorbushina 2007). Fungi, including lichens, can be epilithic (surface dwellers) and/or endolithic (interior dwellers) with *cryptoendoliths* occupying structural cavities, *chasmoendoliths* inhabiting fissures and cracks and euendolithic forms capable of active rock penetration (Cockell and Herrera 2008; Wierzchos et al. 2012). These terms are also used for other rock-inhabiting microorganisms although there may be many overlaps, even for a given species.

### Definition of endoliths (euendoliths)

Endoliths are considered a special category of rock transforming microorganisms. They are defined as those microorganisms which are capable of boring into solid substrates, which contain calcium carbonate and which were manufactured by living organisms (Kohlmeyer 1969; Golubic et al. 2005). The endolithic environment includes the pore spaces in shells and animal skeletons, such as those in corals, and in rocks and the pores between mineral grains and is ubiquitous in all fresh, brackish and salt water bodies, sediments and soil ecosystems. Endolithic boring microorganisms are capable of penetrating many solid substrates especially the calcareous shells of live and dead invertebrate animals as well as calcareous geological formations in extremely stressful habitats. Therefore, these species could be characterised as extremophiles.

### Types of endoliths

Che et al. (1996) identified three ecological groups of the shell (and rock) boring species which attack shells of invertebrates and other calcareous structures: (1) photosynthetic microbial endoliths, (2) heterotrophic microbial endoliths and (3) filter feeding boring invertebrate animals. The early research on endolithic marine planktonic algae was reviewed by Golubic (1969) and the early research on endolithic marine fungi by Kohlmeyer (1969). More recently, Golubic et al. (2005) have updated our knowledge of both groups. Shell boring invertebrate animals are not included his review.

Microbial endoliths include two different lifeforms: ones which colonise existing spaces (endolithic cavities) within solid geological substrates (including porous carbonate and non-carbonated rocks and shells composed of calcium cabonate) and those which actively penetrate (and bore into) these substrates (Golubic 1969).

The photosynthetic (autotrophic) endoliths include a few documented species of Cyanobacteria, Chlorophyta, Phaeophyta and Rhodophyta (Golubic 1969), which live in euphotic zones, and their growth often results in the formation of dark bands with zones of different colour (often green or red) at various depths of the calcareous substrates they occupy. Photosynthetic endolithic communities frequently inhabit the outer few centimetres of rocks exposed at the surface (where light can penetrate). Most of the upper intertidal and supratidal boring phototrophs belong to the Cyanobacteria (Golubic 1969). Photosynthetic microbial endoliths can provide food resources to heterotrophic microbial species nearby. The endolithic phototrophs are protected from parasites, predators and extreme environmental conditions when living inside the calcareous structures.

The heterotrophic microbial endoliths include a few documented parasitic species in the Ascomycota (true fungi) and Labyrinthulomycota (Stramenopilia) (Kohlmeyer 1969; Raghukumar and Lande 1988; Porter and Lingle 1992; Golubec et al. 2005; Raghukumar and Ravindran 2012) and these aggressive species may attack and feed on the photosynthetic endoliths. The heterotrophic endoliths are thought to release proteolytic enzymes which facilitate the breakdown of proteins left over from the process of formation of the shells by the animals. These proteins can provide an additional food resource for the fungi and labyrinthulid endoliths. The fungal hyphae tend to follow the algal filaments and the fibrous proteins as they cross the calcareous structures. The algae presumably do not release proteases and therefore must remain in the parts of the structure, which are composed of only calcium carbonate.

Boring patterns reflect in part the shape and behaviour of the boring microorganisms and in part the structural properties of the shell itself (Che et al. 1996). In the black pearl oyster, the phototrophic endoliths dominated the external prismatic region of the shell whereas the interior nacreous region was attacked mainly by heterotrophs (Che et al. 1996). Infection always begins in the oldest part of the shell.

## Ecological roles of endoliths

We expect that endoliths play important roles in the global calcium carbonate cycle and other ecological processes especially in aquatic ecosystems and may be significantly impacted by global climate change. Endoliths actively contribute to the biodegradation of geological substrates and skeletons and shells of dead animals composed of calcium carbonate. They have been implicated as a causative agent of shell diseases in live molluscs, corals and other phyla of invertebrate animals (Golubic 1969; Kohlmeyer 1969; Che et al. 1996; Golubic et al. 2005; Zuykov et al. 2014). Rates of microbial bioerosion of experimental blocks cut from live skeletons of the coral *Porites* have been estimated at sites along the Great Barrier Reef by Kiene and Hutchings (1994) and Tribollet (2008). The results suggested that endoliths have a significant impact on the overall calcium budget of coral reef ecosystems, but rates were not measured in skeletons or shells of other species or in other ecosystems (Tribollet 2008). A thorough understanding of the ecology of endoliths awaits further investigation into blue carbon research.

## Techniques for observation of endoliths

Historically, endolithic microorganisms have been very poorly studied primarily because they are difficult to detect and correctly identify with standard laboratory procedures and their ecological properties are difficult to study *in vivo* with present technology. Endoliths can be observed often growing *in vivo* on the surface of calcareous structures with the light microscope, but they must be grown in the laboratory in culture to see all stages of their life cycles. They can only be observed growing inside calcareous structures with the light microscope in cast resins or double embedded preparations or with scanning and transmission electron microscopes as chemically fixed specimens using specialised techniques. The specialised techniques for observing endoliths have been reviewed in detail by Golubic et al. (1970, 2005), Porter and Lingle (1992) and Peharda et al. (2015). A method using immunofluorescence has been designed to detect thraustochytrids inside host cells (Raghukumar and Lande 1988). It might be possible to use similar immunofluorescence techniques for studies with other endolithic species.

## Natural history

The natural history of parasitic and saprotrophic endolithic boring microorganisms and the skeletons and shells of the host animals which they inhabit has been well recorded in the fossil record. The significance of endolithic microbial ecosystems in both aquatic and terrestrial ecosystems in general has been reviewed in detail by Walker and Pace (2007).

Fossilised fungal structures have been reported from a variety of mineral substrates including Devonian Rhynie Chert, as fossil lichen mycobionts in stromatolites, in Djebel-Onk phosphorites, Triassic silicified rock, Bitterfield amber and Tertiary Dominican amber (see Burford et al. 2003). Various types of endoliths, including fungi, have been found in marine shells in the Late Ordovician and Middle Devonian volcanic rocks, while microborings have also been found in early Cambrian phosphatic and phosphatised fossils (Taylor et al. 2015). Fossilised microorganisms have also been observed in drilled cores and dredged samples from the ocean floor, with a majority of these findings representing fungi (Schumann et al. 2004; Bengtson et al. 2014). These fungi existed in a symbiotic-like relationship with two types of chemolithotrophic prokaryotes, which appeared to use the structural framework of the mycelium for their growth (Bengtson et al. 2014). Early fossil records of eukaryotes, including fungi and primitive plants in terrestrial ecosystems, appear to have come from the Ordivician period (Heckman et al. 2001). However, it has been postulated that they may have occurred earlier, during the Precambrian, as lichens (Heckman et al. 2001). Fungi do not preserve well in the fossil record, suggesting the possibility of an earlier, unrecorded history (Heckman et al. 2001). Some estimates have placed the origins of the Glomeromycota between 1400 and 1200 MYA, and the separation of Ascomycota and Basidiomycota around 1200 MYA. Some fossils interpreted as early fungi extend back to the Proterozoic (2500–541 MYA) (Taylor et al. 2015). It has been suggested that mycorrhiza-driven weathering may have originated more than 350 MYA, and that it subsequently intensified with the evolution of trees and mycorrhizas (Quirk et al. 2012). Some of the free-living MCF (also referred to as rock-inhabiting fungi) are slowly growing melanised Ascomycetes especially suited to colonising rocks in arid environments. Because these organisms often form early diverging groups in the Chaetothyriales and Dothideomyceta, the ancestors of these two lineages were suggested to be rock-inhabitants. It was deduced that the rock-inhabiting fungi in the Dothideomyceta evolved in the late Devonian, much earlier than those in the Chaetothyriales, which originated in the middle Triassic, both periods correlating with an expansion of arid landmasses. It was proposed that the paleoclimate record provided a good explanation for the diversification of fungi subject to abiotic stresses and adapted to life on rocks (Gueidan et al. 2011).

## Types of substrates and mechanisms for penetration

### Chemistry of rocks

Rocks and minerals represent a vast reservoir of elements and compounds, many of which are essential to life and which must be released in specific soluble forms which can be assimilated by the biota (Burford et al. 2003). These include essential metals as well as nutrients like phosphate. The most common minerals are the silicates, with non-silicates comprising less than 10% of the Earth’s crust, the most abundant of these being carbonates, oxides, sulphides and phosphates. Fungi have been found associated with a wide range of rock types including limestone, soapstone, marble, granite, sandstone, andesite, basalt, gneiss, dolerite, amphibolite and quartz, even from most harsh environments, e.g. hot and cold deserts (Staley et al. 1982; Gorbushina et al. 1993; Sterflinger 2000; Verrecchia 2000). It is likely that fungi are ubiquitous members of the microbiota of all rocks, occurring over a wide range of geographical and climatic zones (Burford et al. 2003). Free-living and symbiotic fungi are therefore associated with elements besides O that account for over 99.9% of crustal rocks (e.g. Na, Mg, Al, Si, P, K, Ca, Ti, Mn and Fe) (Purvis and Pawlik-Skowronska 2008). Fungal activities in rock and mineral transformations can therefore lead to increased mobility of such elements, and other minor crustal components, as well as the formation of secondary mineral products. Fungi can play a role in the dissolution of common minerals including carbonates, phosphates and silicates and less common compounds including oxides and oxalates (Gadd 2010; Gadd et al. 2014; Bindschedler et al. 2016). Lichens are a symbiosis of a fungus with either a cyanobacterium or a trebouxian green algae (see earlier) and actively digest rock surfaces.

### Chemistry and structure of shells and other utilisable calcareous substrates

The fine structure and chemistry of the shells of several species of molluscs have been studied in detail, particularly in the pearl oyster *Pinctada fucata* (Takeuchi and Endo 2006) and the abalone *Haliotis asinina* (Marie et al. 2010). The primary structure, origin and evolution of shell matrix proteins of molluscs have been reviewed by Marin et al. (2008). Oysters have two complex layers in their shells: the nacreous and the prismatic layers (Sudo et al. 1997). Both layers are composed from microlaminate composites of calcium carbonate crystals (aragonite in the nacreous and calcite in the prismatic layer). The major macromolecules are a complex mixture of structural (or matrix) proteins and glycoproteins which determine the framework of each shell layer. The matrix proteins are secreted by shell-forming tissue in the mantle of molluscs. Calcium carbonate crystals grow within the matrix.

## The specific ecological roles of scleractinian corals on coral reefs

Modern corals are a broadly defined group of anthozoan Cnidaria, which grow in shallow tropical and semi-tropical waters in the upper photic zone (Veron 2000). All such corals are a symbiosis between the coral animal (scleractinian or stony corals in the Class Anthozoa) and a dinoflagellate alga (known as zooxanthellae) in the genus *Symbiodinium*, which live in the endoderm (inner layer) of the animal (Veron 2000). Modern research has shown that the *Symbiodinium* species are remarkably diverse, comprising at least eight distinct genetic clades (A–H) (Wham and Lajeunesse 2016), and more are being discovered regularly. Scleractinian corals probably evolved about 250 MYa from rugose corals; and they lay down a skeleton of calcium carbonate aragonite spicules in a process, which is driven by light and dependent on photosynthesis of the zooxanthellae. Corals can broadly be divided into branching and massive forms. The skeletons of corals are finely sculptured and each species has a unique structure by which it can be identified.

Massive corals, such as species of *Porites*, can grow into very large structures, often roughly globose and several meters in diameter. Such massive corals have a thin outer veneer of living coral tissue that encloses an inner mass of largely dead aragonite material. Commonly photosynthetic endoliths occur in this aragonite material. A characteristic green outer layer about 1 cm below the surface of massive corals is formed by species of the green alga, *Ostreobium*, mainly *O. quekettiae* (Gutner-Hoch and Fine 2011). These species of algae are major colonisers (Ralph et al. 2007). In addition, other eukaryotic algae or cyanobacteria can be present in lesser abundance forming distinctive outer layers. Deeper within the coral, inner layers of anoxygenic photosynthetic bacteria are evident by their characteristic absorption spectra. The light absorbing pigments are bacteriochlorophylls rather than chlorophylls, and these bacteria presumably live in the kind of spectral radiation reaching these depths, which is enriched in infra-red radiation that the prokaryotic bacteria but not eukaryotic algae can absorb.

The endolithic microorganisms found inside the skeletons of massive corals fall into the class of organisms that broadly is described as lithoclastic, i.e. they dissolve aragonitic calcium carbonate structures. However, there are other calcifying organisms on coral reefs, most notably the green alga *Halimeda* spp and its relatives. These green algae lay down aragonite, that ultimately forms into grains of calcium carbonate, which constitute over half of the coral sand of lagoon floors (Perry et al. 2016). In addition, the calcite skeletons of calcifying red algae generate a large amount of the calcium carbonate of coral reefs (Anthony et al. 2008). Much less is known about the lithoclastic processes that are responsible for breaking down the green and red algal products. Presumably, endoliths are partly involved as well as surface or interfacial bioeroders.

In addition to those already mentioned, it is becoming increasingly apparent that many other microorganisms on coral reefs interact with calcium carbonate skeletons. Nearly a decade ago, Moore et al. (2008) first reported the isolation of an apicomplexan alga, *Chromera veliae*, from corals. This free-living photosynthetic alga lies on the evolutionary path to non-photosythetic apicomplexans such as the malaria pathogen and is therefore a bridge between photosynthetic phytoplankton, such as the dinoflagellate *Symbiodinium*, and non-photosynthetic, parasitic apicoplexans. Since that time, many more “intermediate” species have been discovered (Keeling 2013); however, their ecological roles on coral reefs are presently poorly understood.

The interaction of environmental factors associated with global climate change (such as temperature increase, ocean acidification, eutrophication, changes in salinity etc.) on coral calcification and the roles of endolithic bioeroders and other organisms in coral ecosystems is very complex (e.g. Diaz-Pulido et al. 2012) and is presently causing very serious ecological damage worldwide (Burge et al. 2013).

It is well established that global warming of the oceans has led to bleaching and the death of corals. This was first established by Hoegh-Guldberg (1999) and recently confirmed beyond doubt by recent events on the Great Barrier Reef, Australia (Hughes et al. 2017). When summer sea-surface temperatures rise to 30°C or above, zoothanxellae are lost from corals. Under severe and prolonged stress, the coral animals starve, and the entire coral bleaches completely and finally dies. Bleaching or loss of colour is caused by loss of photosynthetic pigments. The trigger is connected with inhibition of photosynthesis in the zooxanthellae of the affected coral (Jones et al. 1998). In addition, the increase of greenhouse gases in the atmosphere has led to ocean acidification whereby the upper layers of the oceans have decreased in pH. This has led to a decline in the rate of calcification in corals and other calcifiers on coral reefs. Thus, the effects of global warming and ocean acidification on coral reefs are very serious.

### The mechanisms of rock and mineral transformations

Fungal colonisation of rock and mineral substrates can result in physical and biochemical effects which are influenced by substrate chemistry and mineralogy. The presence of weatherable minerals such as feldspars and clays may increase susceptibility to attack (Warscheid and Braams 2000). Typical transformation mechanisms involve physical and biochemical processes that are generally interlinked (Gadd 2017). Physical mechanisms include penetration by the hyphae along points of weakness, or direct tunnelling or boring, especially in weakened or porous substrata (Jongmans et al. 1997; Hoppert et al. 2004). Fungal tunnels within soil minerals have been explained as a result of dissolution and “burrowing” within the mineral (Jongmans et al. 1997). Tunnels may also result after fungal exploration of pre-existing cracks, fissures and pores in weatherable minerals and formation of a secondary mineral matrix of the same or different chemical composition as the substrate, e.g. secondary CaCO_3_ or an oxalate (Fomina et al. 2010). This can result in the fissures and cracks becoming cemented with mycogenic minerals, and after death and degradation of fungal hyphae, tunnels are left within the minerals. There is some debate as to the relative significance of fungal boring or tunnelling as compared to penetration through pores and points of weakness. However, it would seem unusual if fungi were not capable of direct boring, a feature found in many groups of microorganisms (Cockell and Herrera 2008). Fungi possess the properties of filamentous apical growth, cell turgor pressure and the ability to dissolve minerals that make it possible for fungi develop such a pattern of growth. Tunnelling by fungi has been observed clearly for some natural biogenic minerals such as ancient ivory (Pinzari et al. 2013). Weakening of a mineral lattice can also occur through wetting and drying cycles and expansion or contraction of the biomass. Lichens can cause mechanical damage due to penetration by their anchoring structures, composed of fungal hyphae (Chen et al. 2000). Biochemical weathering of rock and mineral substrates occurs through excretion of hydrogen ions, CO_2_, organic acids, siderophores and other metabolites, for example, resulting in pitting, etching or dissolution (Gadd 2010). Some organic metabolites affect dissolution by complexing with constituent metals, permitting removal of the mineral in a mobile form. Biogenic organic acids are very effective in mineral dissolution and are one of the most damaging agents affecting mineral substrates (Gadd 2007). Of the suite of organic acids produced by fungi, oxalate is of major significance through metal complexation and dissolution effects as well as causing physical damage by formation of secondary metal oxalates expanding in pores and fissures (Gadd et al. 2014). Citric and gluconic acid are other significant fungal metabolites. MCF can mediate “micropitting” in rocks leading to cavities that contain the fungal colonies. This appears to result from mechanical destruction caused by cell turgor pressure and extracellular polysaccharide production rather than the acid dissolution caused by many other fungi (Marvasi et al. 2012). These fungi may also form casual mutualistic associations with algae in rock crevices (Gorbushina 2007).

### The mechanisms of penetration into calcareous structures

Endoliths which bore into solid substrates possess specialised filaments which actively penetrate readily soluble substrates, most commonly composed of calcium carbonate, such as calcareous rocks, shells of molluscs and corals, skeletal fragments and sand grains. When microbial endoliths actively penetrate calcarious substrates, they leave characteristic boring traces on the surface. It is thought that endoliths release substances, possibly organic acids (as discussed previously), which can dissolve calcium carbonate.

Microbial parasites must bore through the matrix proteins to penetrate mollusc shells presumably by excreting extracellular proteases. Bédouet et al. (2007) detected several protease inhibitors in the water-soluble organic matrix in nacre from the oyster *Pinctada margaritifera*. These proteases inhibitors were active against some serine and cysteine proteases. Bédouet et al. (2007) proposed that these protease inhibitors might play a role in defence of host mollusc species against microbial parasites.

## General comments/conclusions

Endoliths are important components of the marine calcium carbonate cycle because they actively contribute to the biodegradation of shells of dead animals composed of calcium carbonate and calcareous geological substrates. They have been implicated as a causative agent of shell diseases in live corals, molluscs and other invertebrate animals which have shells composed of calcium carbonate (Golubic 1969; Kohlmeyer 1969; Che et al. 1996; Golubic et al. 2005; Zuykov et al. 2014).

Endolithic microorganisms have important roles as saprotrophs in bio-erosion of many calcium carbonate substrates, as parasites on the production of commercially important animal species, regulate biodiversity in marine ecosystems, and they respond to environmental factors which are involved significant components of global climate change.

Heterotrophic endoliths can destroy the shells of animal species living in marine ecosystems or bioerode dead shells buried in the sediment (Kendrick et al. 1982; Raghukumar and Lande 1988). The dissolution of calcium carbonate in bioerosion causes the release of carbon dioxide into the marine environments, which increases acidification. Calcareous substrates contain large amount of carbon. Therefore, heterotrophic endoliths are key players in the marine calcium carbonate cycle.

The total amount of global calcareous substrates in sediments in the ocean has not been accurately estimated. However, as carbon dioxide from bioerosion of calcium carbonate in the ocean eventually enters the atmosphere, large losses in calcareous substrates in carbon sinks would be expected to result in increased heat retention by the atmosphere, increasing global mean temperatures. If rising temperatures and acidity in the ocean increase the rate of growth of endolithic fungi, this could provide a positive feed-back mechanism potentially accelerating the rate of climate change.

Many studies suggest that the prevalence of emerging infectious diseases is currently increasing in all ecosystems including coral reefs (e.g. Fisher et al. 2012; Burge et al. 2013). Heterotrophic fungal endoliths comprise a group which contains parasites and which can cause diseases in corals. Since fungal endoliths are present in both healthy and diseased corals, they are considered to be opportunist parasites, which can cause disease but only under certain environmental conditions. Probably stress, depression of immune responses and global climate change factors are all important in triggering disease. Nonetheless, if emerging infectious disease causes large losses in population sizes and in biodiversity, the lost carbon will undoubtedly enter the atmosphere as carbon dioxide produced by respiration and further contribute to global climate change.
